# δ-Subunit Containing GABA_A_ Receptors Modulate Respiratory Networks

**DOI:** 10.1038/s41598-017-17379-x

**Published:** 2017-12-22

**Authors:** Gaspard Montandon, Haiying Wu, Hattie Liu, Michael T. Vu, Beverley A. Orser, Richard L. Horner

**Affiliations:** 1 Keenan Research Centre for Biomedical Science, St. Michael’s Hospital, Toronto, Canada; 2 Department of Medicine, University of Toronto, Toronto, Canada; 3 Department of Physiology, University of Toronto, Toronto, Canada; 4 Department of Otolaryngology, The Second Affiliated Hospital of Kunming Medical University, Kunming, China; 5 Department of Anesthesia, University of Toronto, Toronto, Canada; 6 Department of Anesthesia, Sunnybrook Health Sciences Centre, Toronto, Canada

## Abstract

Persistent and stable respiratory activity across behavioral states is key to homeostasis. Extrasynaptic δ-subunit containing GABA_A_ receptors (δGABA_A_Rs) mediate tonic inhibition and regulate network activity. However, the influence of δGABA_A_Rs on respiratory rhythm and motor outputs is unknown. We manipulated extra-synaptic GABA_A_ receptor function in the preBötzinger Complex (preBötC), a site central to the generation of inspiratory motor activity in mammals. Activation of preBötC δGABA_A_Rs in anesthetized rats and wild-type mice decreased breathing rate. In δGABA_A_R knockout (*Gabrd* ^−/−^) mice, however, δGABA_A_Rs activation had no effect on breathing rate. We then found that during active wakefulness associated with behaviors and movements, diaphragm activation was higher in the *Gabrd* ^−/−^ compared to wild-type mice, but not in other states. These findings identify that δGABA_A_Rs modulate the respiratory network, which is critical to understand how δGABA_A_Rs change breathing in pathological conditions affecting extra-synaptic GABA_A_ receptor function such as exposure to anesthetics and neurosteroids.

## Introduction

Rhythmic breathing is generated by a complex neural network in the brainstem^1^ that combines excitatory and inhibitory circuits. The respiratory network produces an intricate respiratory pattern with three phases: inspiration, post-inspiration, and late expiration. Inspiratory motor output is driven by a population of neurons that forms the preBötzinger Complex (preBötC)^2–4^, whereas expiratory activity is regulated by the Bötzinger Complex^5^, a structure with direct projections from and to the preBötC, and the retrotrapezoid nucleus^6^. In the preBötC, excitatory neurons drive inspiratory output^2^, whereas inhibitory neurons coordinate the generation of inspiratory and expiratory phases^7^. Optogenetically targeted excitation of glycinergic neurons in the preBötC shape respiratory pattern by inducing prolonged apnea, but rhythm persists even after substantial inhibition of glycinergic cells^8^. The functional role of GABAergic neurons in the preBötC is, however, not clear. Expression of the GABA-synthesizing enzyme glutamate decarboxylase shows that GABAergic neurons constitute a large part of inhibitory preBötC neurons^9^. In brainstem sections *in vitro*, blocking GABAergic neurotransmission increased rhythm variability, but did not otherwise change the frequency of rhythmic bursts^10^. *In vivo*, application of the GABA_A_ receptor agonist muscimol at the preBötC abolished rhythmic activity^5^, whereas the GABA_A_ receptor antagonist bicuculline did not change rhythmic breathing, but suppressed the Breuer-Hering inspiratory inhibitory reflex^11^. In rabbits, GABA_A_ receptor blockade in the preBötC decreased respiratory rate, but did not completely blocked it^12^. These studies suggest that the endogenous role of GABA_A_ receptors in the preBötC is to shape respiratory motor output, for instance, during voluntary control of breathing^11^, but this hypothesis has not yet been tested.

GABA_A_ receptors mediate fast synaptic inhibition, whereas GABA can also activate highly-sensitive extra-synaptic GABA_A_ receptors and produce tonic inhibition^13^. Extrasynaptic δ-subunit containing GABA_A_ receptors (δGABA_A_Rs) mediate tonic inhibition and regulate network excitability^14^ and outputs^15^. δGABA_A_Rs also function to dampen network excitability and prevent excessive excitation^16^. In the context of endogenously active rhythmic networks, such as the respiratory network, these observations suggest that extra-synaptic δGABA_A_Rs may also play a significant role in modulating respiratory activity. However, the role of extra-synaptic δGABA_A_Rs in modulating respiratory rhythm has never been tested. Here, we propose that δGABA_A_Rs provide a level of tonic inhibition in the respiratory network to modulate rhythmic breathing. To test these concepts, we pharmacologically manipulated the preBötC of rats and mice, the latter including wild-type mice and mice lacking δGABA_A_Rs^17^. Our data identify for the first time that δGABA_A_Rs function to modulate respiratory network activity.

## Methods

### Experimental animals

All procedures were performed in accordance with the recommendations of the Canadian Council on Animal Care, and were approved by the University of Toronto Animal Care Committee. Twelve adult male Wistar rats (255–350 g, Charles River, Saint-Constant, Quebec, Canada), and nineteen adult mice lacking δGABA_A_Rs (*Gabrd* ^−/−^) plus nineteen wild-type littermate controls were used for physiological recordings (body weight: 30.4–37.0 g). The generation of *Gabrd* ^−/−^ mice has been described previously^18^. Mice and rats were housed with free access to food and water under a 12-hour light:dark cycle (lights on at 7 am).

### Microperfusion and recordings in anesthetized rats

In anesthetized rats, we used reverse-microdialysis to unilaterally microperfuse selected agents into the preBötC. The experimental procedures were as described previously^19,20^. Briefly, we recorded activities of the diaphragm and tongue muscles in isoflurane-anesthetized (2–2.5% inspired), tracheotomised and spontaneously breathing (50% inspired oxygen, balance nitrogen) rats. Diaphragm muscle activity was recorded using stainless steel bipolar electrodes positioned and sutured onto the right crural diaphragm. Tongue muscle activity was recorded using two stainless steel needles inserted via a per-oral approach. The electromyogram signals were amplified (CWE Inc., Ardmore, Pennsylvania, USA), band-pass filtered (100–1000 Hz), integrated, and digitized at a sampling rate of 1000 Hz using an acquisition system and Spike software version 6 (both from Cambridge Electronic Design Limited, Cambridge, UK). Rats were kept warm with a heating pad during the experiments. Using a dorsal approach, a microdialysis probe (CX-I-12-01, Eicom, Kyoto, Japan) of 200 µm diameter and 1 mm diffusing membrane length was inserted into the preBötC using a stereotaxic frame and micromanipulator (ASI Instruments, Warren, Michigan, USA). The probe was placed 12.2 mm posterior, 2.0 mm lateral, and 10.5 mm ventral to bregma according to a standard rat brain atlas^21^ and our previous experience with this model^19,20^. Baseline (aCSF) data for breathing rate and diaphragm amplitude were averaged over a 30-min period preceding the start of THIP infusion. THIP data were averaged for a 30-min period starting 30 min after THIP infusion was initiated. Only experiments where the probe was located within 2 mm from the center of the preBötC were analysed.

### Anatomical location of the microdialysis probes in rats

As described previously we used three criteria to target the preBötC, to position the probe, and to confirm its location^19,20^: (i) When the probe was inserted into the medulla, tongue muscle activity decreased ~30% as it reached the vicinity of the preBötC. (ii) Microperfusion of the µ-opioid receptor agonist DAMGO (5 µM) decreased breathing rate by approximately 50%^19^. (iii) Post-mortem histology confirmed the probe location in the preBötC using anatomical markers such as the semi-compact division of nucleus ambiguus and the caudal part of the facial nucleus. We used the caudal part of the facial nucleus as a reference to identify the brain section located 11.6 mm posterior to bregma according to Paxinos atlas^21^. Once the coronal section where the probe was located was identified, we used the nucleus ambiguus to then determine the medial-lateral and dorsal ventral coordinates. Once the location of the probe site was determined, we determined 3-dimensional coordinates (anterior-posterior, medial-lateral, and dorsal-ventral) using bregma as reference. We used these three anatomical and functional criteria, and experience from our previous studies, to confirm that the probes were positioned in the region of the preBötC. On rare occasions (~1/20 experiments), the probes damaged the preBötC and respiratory rhythm was irregular and unstable. In such an event, we discontinued the experiment.

### Microperfusions

Fresh artificial cerebrospinal fluid (aCSF) was made for each experiment with the following composition (in mM): NaCl (125), KCl (3), KH2PO4 (1), CaCl2 (2), MgSO4 (1), NaHCO3 (25), and glucose (30); pH was adjusted to 7.4 by bubbling CO_2_ into aCSF. A microdialysis probe was perfused with aCSF and baseline levels of the physiological variables were recorded for at least 30 min followed by 120 min of recordings during microperfusion of the selected agents. We applied the δGABA_A_R-preferring agonist 4,5,6,7- tetrahydroisoxazolo[5,4-c]pyridin-3-ol hydrochloride (THIP), the GABA_A_ receptor antagonist bicuculline and the µ-opioid receptor antagonist DAMGO to the preBötC. All drugs were obtained from Tocris (Minneapolis, Minnesota, USA).

### Correlation between the distance from perfusion sites to the preBötC and the resultant effect on respiratory activity

We calculated the correlation to relate the location of the intervention sites with the resultant effect on respiratory activity as previously described^19^. The rationale for these correlations is that for a locus of effect of THIP at the preBötC, the latency for the drug to diffuse through the tissue and to progressively change respiratory activity will vary as a function of the distance of the probe from the preBötC. We first determined the locations of the intervention (perfusion) sites using the criteria described above. For each animal, we also calculated the distances from the corresponding perfusion site to the center of the preBötC (coordinates: anterior-posterior = −12.2 mm, dorsal-ventral = −10.5 mm, medial-lateral = −2.0 mm). We then calculated the latencies to a 10% change in respiratory rate in response to THIP, and correlated these distances with the latencies or the magnitudes of THIP effects on respiratory rate.

### Microperfusion and recordings in anesthetized mice

In anesthetized (isoflurane, 1.5–2%), spontaneously breathing (50% oxygen, balance nitrogen) wild-type and *Gabrd* ^−/−^ mice, we also used reverse microdialysis to microperfuse agents into the preBötC. We recorded diaphragm muscle activity using a similar approach to the rats and as previously described for mice^22^. The mice were also kept warm with a heating pad. We inserted the microdialysis probe into the medulla 6.7 mm posterior, 1.2 mm lateral, and 5.7 mm ventral to bregma according to the standard brain atlas^23^ and prior experience^24^. For wild-type or *Gabrd* ^−/−^ mice, baseline levels were recorded for at least 30 min followed by 120 min of recordings during microperfusion of the selected agents. Baseline (aCSF) mean data were calculated for the 30-min preceding the start of THIP infusion. THIP mean data were calculated between 30–60 min after the start of THIP infusion. The anatomical and functional criteria defined in rats were also used for the experiments in mice. We used the caudal part of the facial nucleus to identify the brain section 6.5 mm posterior to bregma as shown in the mouse Paxinos atlas^23^. All experiments with probe sites located within 1 mm were used to calculate mean data.

### Implantations for chronic interventions

To identify the role of δGABA_A_Rs in regulating respiratory and motor activities in freely behaving wild-type and *Gabrd* ^−/−^ mice, we recorded electroencephalogram (EEG) and postural (neck) muscle electrodes to identify sleep-wake states, and diaphragm muscle electrodes for respiratory muscle recordings. One week prior to the behavioural experiments, sterile surgery was performed under isoflurane anaesthesia to implant the mice with the EEG, neck, and diaphragm muscle electrodes. To record diaphragm activity, two wires were sutured onto the costal diaphragm via an abdominal approach. The mouse was placed in the prone position in the stereotaxic apparatus (Model SAS-4100) with blunt ear bars, and three holes were drilled into the skull for the placement of the EEG electrodes. Two stainless steel screws (size 0–80 × 1/16, Plastic One Inc., Roanoake, VA, USA) were placed approximately 1 mm to the right and 1 mm anterior to bregma, and 1 mm to the left and 2 mm posterior to bregma for EEG activity; the third electrode used as a common reference was placed 2 mm to the left and 2 mm anterior to bregma. Insulated multi-stranded stainless steel wires were also sutured on the dorsal neck muscles to record the electromyogram. Post-surgical care consisted of a subcutaneous injection of an anti-inflammatory drug (ketoprofen, 2 mg/kg) and an analgesic (buprenorphine, 1 mg/kg).

### Recordings in freely behaving mice

Mice recovered for one week prior to the experiments. On the first and second experimental days, mice were connected to the recording apparatus and placed in a large open-topped Plexiglas bowl filled with fresh bedding, food and water. Mice were left in the chamber for about 6 hours each day to habituate the animal to its environment. On the third day, experiments were performed to record electrophysiological signals while the mice were awake and asleep, and able to move freely. The Plexiglas bowl was placed on a rotating turntable (Raturn, BASi, West Lafayette, IN, United States) that automatically adjusts its position when the mouse physically moves; this response of the turntable avoids entanglements of the recording cable. The movement of the turntable were also recorded as a DC voltage. EEG, neck, and diaphragm muscle activities were recorded for a period of 3 hours from 10am to 1 pm. Data were amplified, filtered, moving-time averaged, sampled and analyzed as described previously^19^.

### Identification of sleep-wake states

For every 10-second epoch, sleep-wake states were visually identified as active wakefulness (AW), quiet wakefulness (QW), non-rapid-eye-movement (NREM), and rapid-eye-movement (REM) sleep. Prevailing sleep/wake states were identified according to standard criteria^25^. EEG frequencies were also calculated in the following frequency bands: δ (1–4 Hz), θ (4–7.5 Hz), α (7.5–13.5 Hz), β_1_ (13.5–20 Hz), β_2_ (20–30 Hz). AW was characterized by low δ frequencies, high neck muscle activity and was often accompanied by body movements. QW was characterized by low δ frequencies, and low neck muscle activity without body movements. NREM sleep was characterized by high δ frequencies, high EEG amplitude and low neck muscle activity. REM sleep was characterized by low δ frequencies, high θ frequencies and low neck muscle activity.

### Behavioural assessments and movements

To determine whether the animal was moving in its environment, we used a combination of video analysis, activation of the rotating table, and activation of postural neck muscle. Specifically, we separated periods of active wakefulness into two distinct periods for subsequent comparison with each other and with quiet wakefulness: (i) Periods of active wakefulness with body movements (i.e. MOV). These periods were recorded during the automatic adjustments of the turntable under the recording chamber as the mouse physically moved, and were verified by video recordings. (ii) Active wakefulness without such movements (i.e. NO-MOV). Such periods of NO-MOV occurred during specific behaviors while stationary, such as sniffing or grooming, and were also identified from the video recordings. (iii) Periods of quiet wakefulness as identified by low δ EEG frequencies, low neck muscle activity and no body movements.

### Diaphragm and neck muscle measurements

Breathing rate was defined as the number of diaphragmatic breaths per minute. Diaphragm amplitude was defined as the amplitude of integrated diaphragm of a breath by subtracting the lower diaphragm signal to the peak diaphragm signal. To determine synchronization of postural neck muscle and diaphragm muscle activities, we computed the cross-correlation function using MATLAB Signal Processing Toolbox (function *xcorr* MATLAB, R12, Mathworks). This function is a measure of similarity of two waveforms. A value of 1 means that the two signals are identical, whereas a value of 0 means that they are completely different. Cross-correlation function was calculated for each 10 s epoch and associated with a specific state. Breathing rates, diaphragm amplitude and neck muscle activities were also measured for each 10-second epoch.

### Statistical analysis

In the figures, group data are illustrated as the mean ± standard error of the mean (S.E.M.). Before analysis of variance tests are performed, all data are first tested for normality with the Shapiro-Wilk test and for equality of variances with the Brown-Forsythe test. For the studies in rats, we used 1-way ANOVAs with the repeated factor being the treatment with aCSF or drug of choice. For the studies in mice, we tested for group differences using 2-way ANOVAs, with one factor being genotype (i.e. wild-type or *Gabrd* ^−/−^) and the second repeated factor being drug treatment (i.e. aCSF or drug of choice). If the ANOVA was statistically significant, an all pairwise multiple comparison procedure (Holm-Sidak tests) was then used to determine significant differences between conditions (e.g., aCSF versus drug). In studies with small groups of animals (n = 6), the non-parametric Mann Whitney rank sum test was used to test significance. All statistical tests were two-tailed with the level of significance set at *P* < *0.05*. All tests were performed with SigmaPlot version 11 (Systat Software Inc, San Jose, CA, USA).

## Results

### Activation of δGABA_A_Rs at the preBötC reduces rhythmic respiratory activity

**Figure 1:**
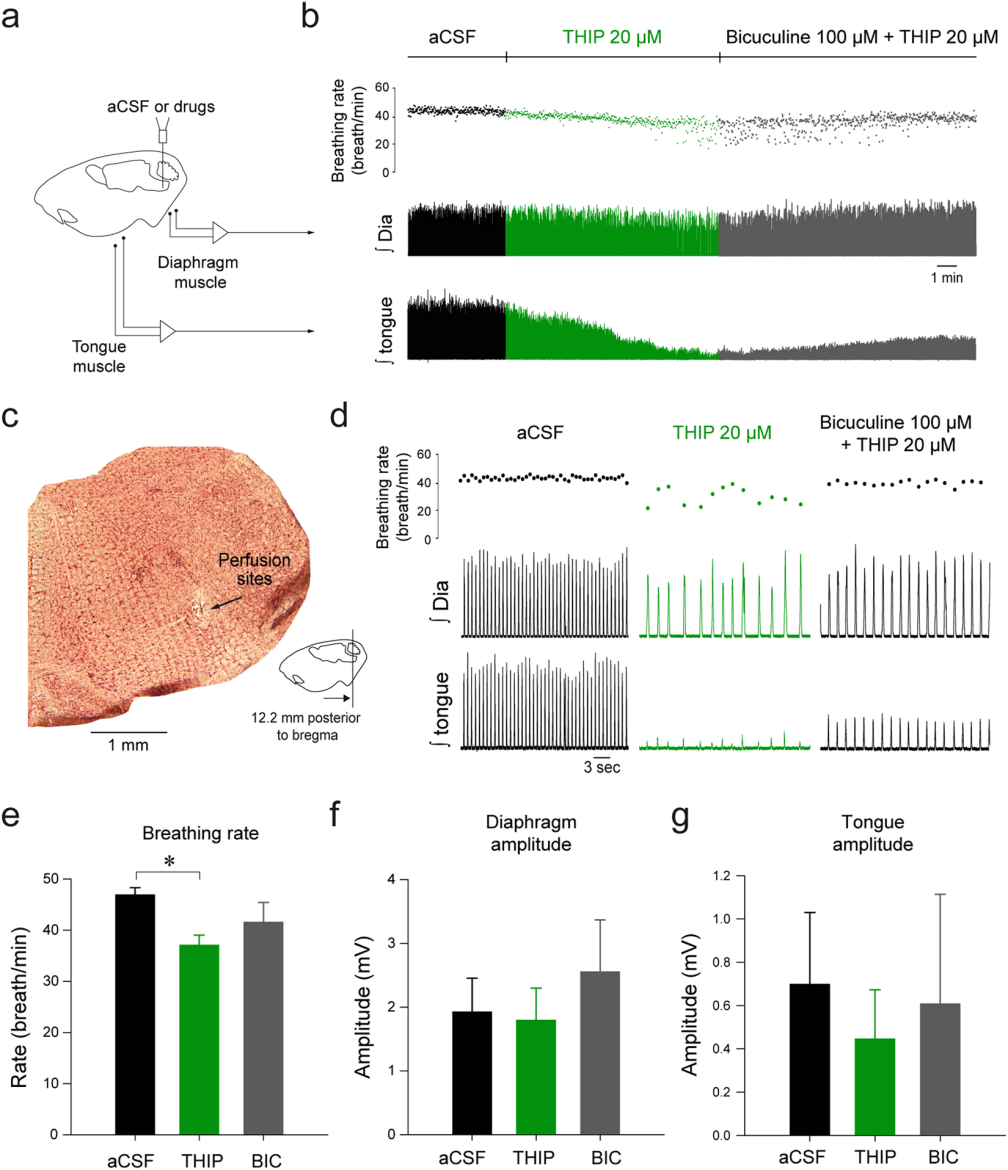
Activation of δGABA_A_Rs with the δGABA_A_R-preferring agonist THIP slows rhythmic breathing. In the anesthetized rats, THIP was microperfused in the preBötzinger Complex while diaphragm and tongue muscles were recorded (**a**). THIP (20 µM) substantially decreased breathing rate (**b,d**), an effect that was partially reversed by addition of the GABA_A_ receptor antagonist bicuculline (100 µM). Microperfusion was done in the preBötzinger Complex region (**c**). Mean data indicate that THIP significantly reduced breathing rate (**e**) but not diaphragm muscle (**f**) and tongue muscle activities (**g**). Data are indicated as mean ± SEM. *Indicates mean data significantly different from aCSF condition with P < 0.05). Dia, diaphragm muscle. aCSF, artificial cerebro-spinal fluid. THIP, 4,5,6,7-Tetrahydroisoxazolo[5,4-c]pyridin-3-ol hydrochloride. BIC, bicuculline.

Tonic inhibition mediated by extrasynaptic GABA_A_ receptors regulates the magnitude and frequency of oscillations in neuronal networks by adjusting the excitability of the network^13^. Here we tested this concept in the respiratory network and determined the contribution of δGABA_A_Rs to rhythmic breathing. To activate δGABA_A_Rs, we first applied the δGABA_A_R-preferring agonist THIP to the preBötC of anesthetized rats while simultaneously recording diaphragm and tongue muscle activities. Microperfusion of THIP (20 µM) into the preBötC (Fig. 1a–c), at a concentration known to activate δGABA_A_Rs^26^, decreased breathing rate and tongue muscle activity, but did not affect diaphragm amplitude (Fig. 1b,d). The GABA_A_ receptor antagonist bicuculline partially reversed the reduction induced by THIP (Fig. 1b,d). Only experiments where the microdialysis probe was located within 2 mm from the center of the preBötC were analysed. According to this criteria, 3 out of 12 animals were excluded. Group mean data from 9 animals show that THIP significantly decreased breathing rate by 23.2 ± 2.1% (n = 9, *P* < *0.001*, Fig. 1e) but not diaphragm amplitude (*P* = *0.122*, Fig. 1f). Tongue muscle activity was not significantly changed (*P* = *0.210*, Fig. 1g).

**Figure 2:**
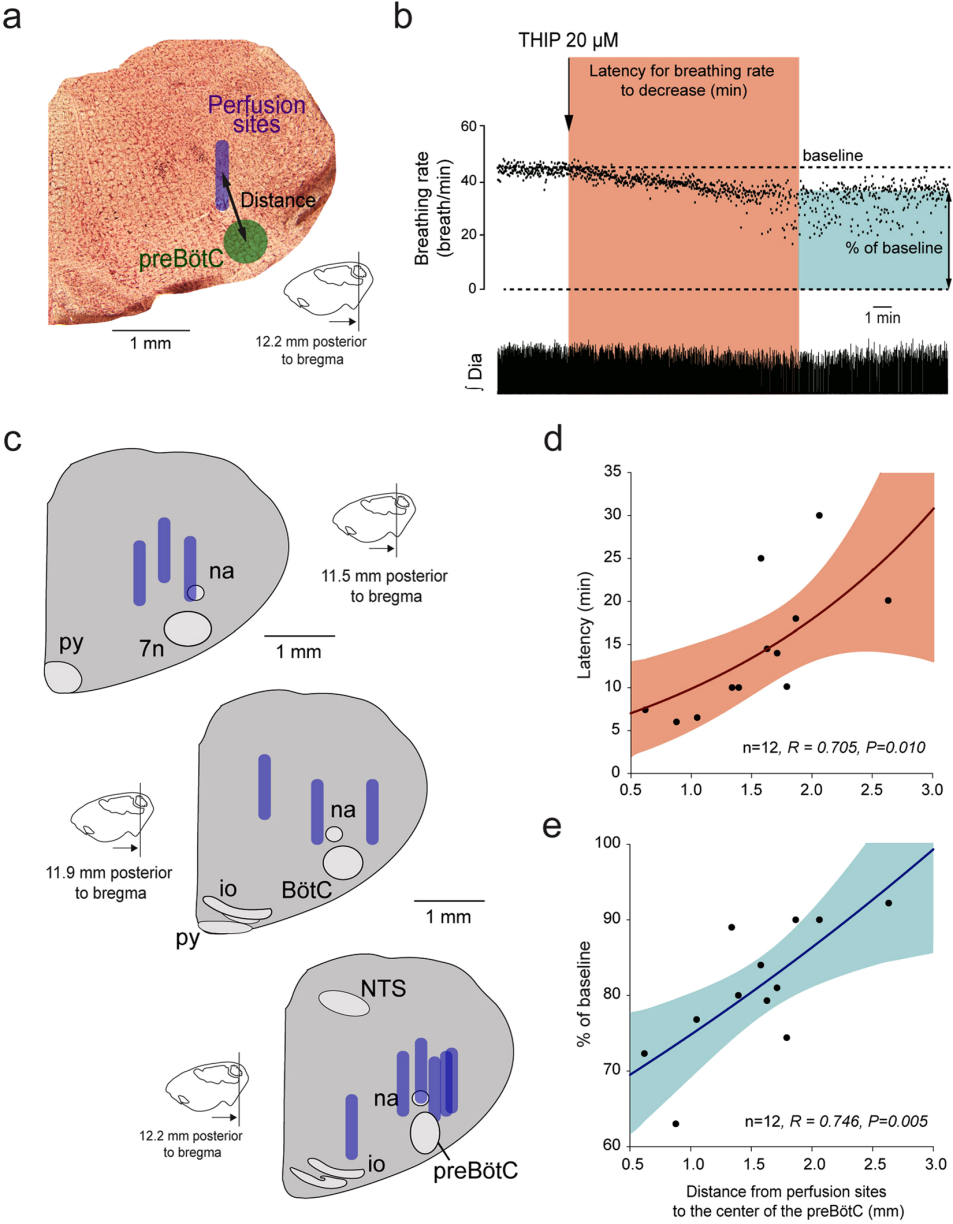
WCMicroperfusions of THIP in the medulla were performed and the location of the perfusion sites were determined by histology (**a**). The distance from the perfusion sites to the center of the preBötzinger was calculated for each animal. Using diaphragm recordings, the duration from the start of microperfusion to a 10% decrease of breathing rate was calculated. Red indicates the latency to diffuse through the tissue and decreased breathing rate by 10%. Blue indicates percentage of baseline rate due to THIP after 30 min of drug perfusion (**b**). Diagrams showing brainstem sections where the microdialysis membranes (indicated by purple bars) were positioned for each experiment (**c**). Positive and significant correlation between latencies and distances from perfusion sites to the preBötC (**c**, n = 12) and between percentages of baseline breathing rate and distances from perfusion sites to the center of the preBötC (**d**, n = 12). Dia, diaphragm muscle. THIP, 4,5,6,7-Tetrahydroisoxazolo[5,4-c]pyridin-3-ol hydrochloride.

To identify whether the preBötC is sensitive to the δGABA_A_R-preferring agonist THIP, we determined the relationship between the proximity of the perfusion sites (Fig. 2a) to the preBötC and the latency at which THIP reduced breathing rate^19^ (Fig. 2b). The reasoning is that when THIP is microperfused close to the preBötC, then the latency for the agent to decrease breathing rate will be short, whereas when the microperfusion is further away from the preBötC the latency will be longer. We calculated the correlations of the *distances* (Fig. 2a) from perfusion sites (Fig. 2c) to the center of the preBötC with: (i) the *latencies* for THIP to decrease breathing rate by 10%, and (ii) the *magnitudes* of the reductions in breathing rate (Fig. 2b). We identified significant correlations between the *distances* and (i) the *latencies* of the breathing rate reductions (R = 0.705, *P* = *0.010*, n = 12, Fig. 2d) as well as (ii) the *magnitudes* of the reductions in breathing rate (R = 0.746, *P* = *0.005*, Fig. 2e). These results show that when THIP is microperfused close to the preBötC it quickly and markedly reduced breathing rate, with the responses being slower and of lesser magnitude with interventions performed further away from the preBötC.

### δGABA_A_Rs inhibit rhythmic breathing in wild-type but not *Gabrd*^−/−^ mice

**Figure 3:**
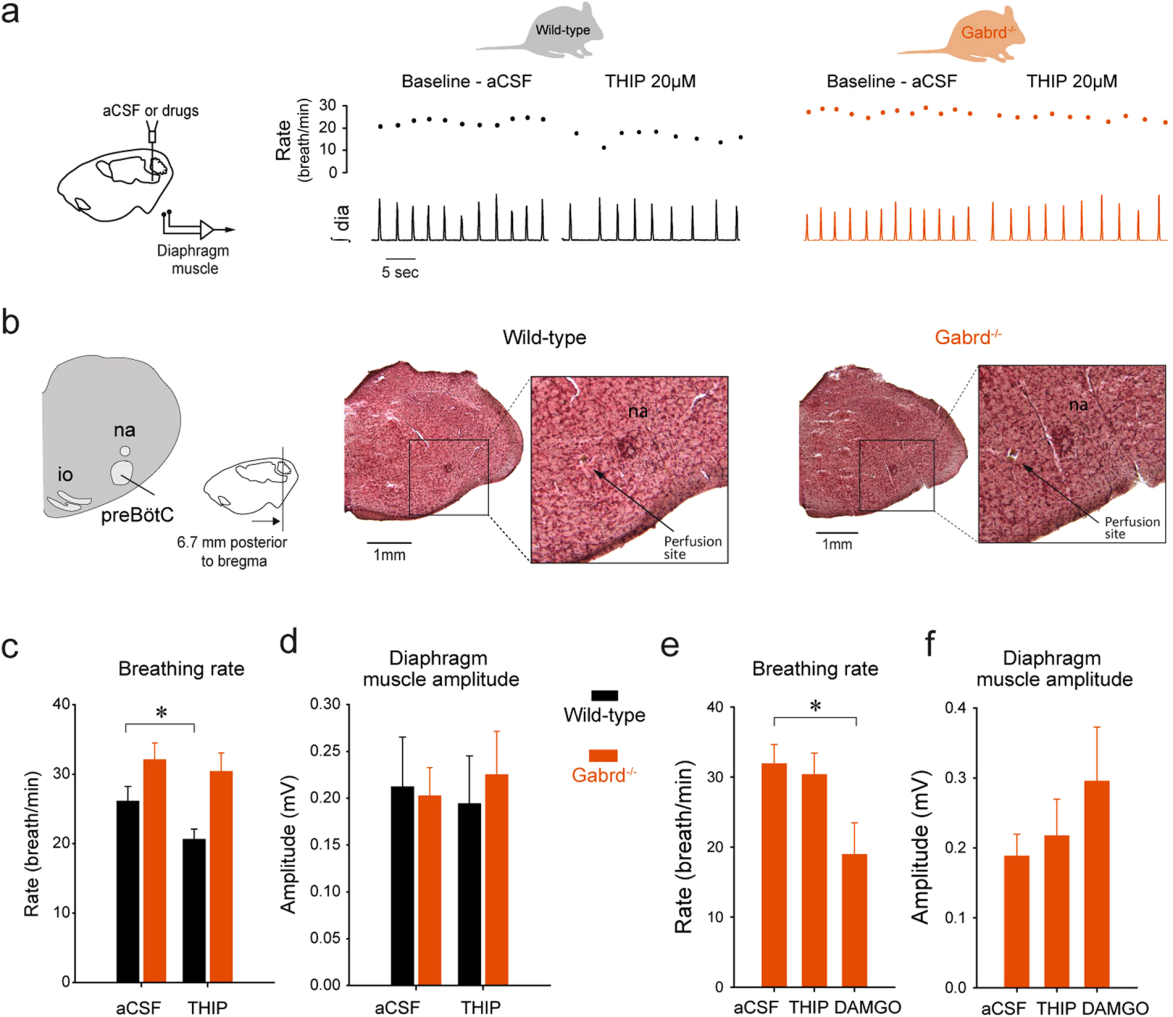
THIP decreases breathing rate by acting on δGABA_A_R. In anesthetized mice, THIP was microperfused in the preBötzinger Complex while diaphragm muscle was recorded (**a**). THIP (20 µM) substantially decreased breathing rate. In *Gabrd* ^−/−^ mice, THIP did not affect breathing rate (**c**). Post-mortem histology showed that microperfusion was performed in the vicinity of the preBötC in both wild-type and *Gabrd* ^−/−^ (**b**) mice. Comparison of wild-type and *Gabrd* ^−/−^ (**c**) showed that THIP significantly reduced breathing rate in wild-type, but not in *Gabrd* ^−/−^ mice. THIP had no effect on diaphragm muscle amplitude both in wild-type and *Gabrd* ^−/−^ mice (**d**). As positive control, the µ-opioid receptor agonist DAMGO (10 µM) was microperfused in *Gabrd* ^−/−^ mice to determine whether the probe was correctly positioned. DAMGO consistently decreased breathing rate (**e**) to the level found with THIP in wild-type mice, but did not change diaphragm amplitude (**f**). Data are indicated as mean ± SEM. *Indicates mean data significantly different from corresponding aCSF condition with P < 0.05. Dia, diaphragm muscle. aCSF, artificial cerebro-spinal fluid. THIP, 4,5,6,7- Tetrahydroisoxazolo[5,4-c]pyridin-3-ol hydrochloride.

Although THIP is δGABA_A_R-preferring agonist^27^, we further verified that THIP selectively targets δGABA_A_Rs by using *Gabrd* ^−/−^ mice that lack these receptors. We microperfused THIP into the preBötC region of anesthetized wild-type and *Gabrd* ^−/−^ mice (Fig. 3a). Example (Fig. 3b) and group data (Fig. 3c) show that microperfusion of THIP (20 µM) into the preBötC region decreased breathing rate in the wild-type but not *Gabrd* ^−/−^ mice. Two-way repeated measures ANOVA showed that THIP significantly reduced breathing rate, but that the effect depended on genotype (*P* = *0.032*, n = 8 per group). Post-hoc analyses identified a decrease in breathing rate of 23.2 ± 2.6% in the wild-type mice (*P* < *0.001*, Fig. 3c), but no changes in *Gabrd* ^−/−^ mice (*P* = *0.155*, Fig. 3c). Responses were specific to breathing rate as there were no THIP-induced changes in diaphragm amplitude in the wild-type or *Gabrd* ^−/−^ mice (*P* = *0.130*, Fig. 3d). To determine whether the preBötC region was sensitive to other manipulations not involving δGABA_A_Rs, i.e., that the absence of effects in the *Gabrd* ^−/−^ mice was not due to unresponsive neurons or incorrectly positioned probes, we perfused the µ-opioid receptor agonist DAMGO (10 µM) into the preBötC region^19^ of *Gabrd* ^−/−^ mice. DAMGO activates µ-opioid receptors and decreased breathing rate by 41.9 ± 3.6% (*P* = *0.016*, ANOVA on ranks, Fig. 3e), but did not change diaphragm amplitude (*P* = *0.486*, Fig. 3f), therefore showing that microperfusion of DAMGO was performed in the responsive preBötC region and had the capacity to inhibit breathing rate by activating µ-opioid receptors as previously demonstrated^22^.

### Activation of δGABA_A_Rs following systemic injection of THIP decreases rhythmic breathing

**Figure 4:**
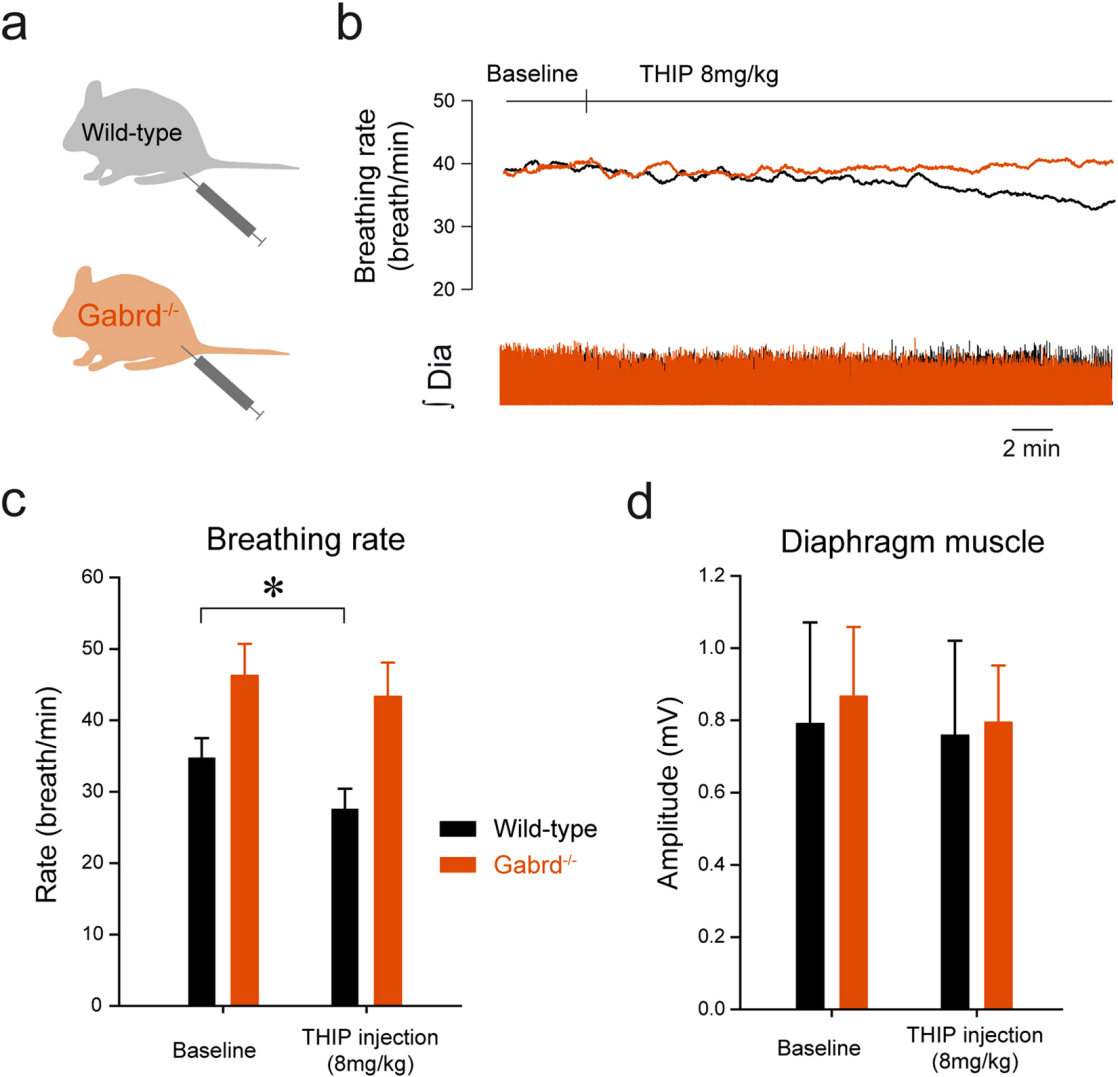
Systemic activation of δGABA_A_R decreases rhythmic breathing in anesthetized wild-type, but not in *Gabrd* ^−/−^ mice. Diaphragm muscle activity was recorded during intramuscular injection of THIP in wild-type (n = 6) and *Gabrd* ^−/−^ (n = 6) mice (**a**). THIP (8 mg/kg) significantly decreased breathing rate in wild-type but not in *Gabrd* ^−/−^ mice (**b,c**). THIP did not change diaphragm muscle amplitude (**d**). Dia, diaphragm muscle activity. *Indicates mean significantly different from the corresponding baseline mean with *P* < *0.05*. Data are indicated as mean ± SEM.

To determine whether general activation of δGABA_A_Rs would also affect respiratory activity, we systemically administered THIP in anesthetized mice while recording diaphragm muscle activity (Fig. 4a,b). Example (Fig. 4b) and group data (Fig. 4c) identified that the systemic injection of THIP (8 mg/kg) decreased breathing rate in the wild-type but not *Gabrd* ^−/−^ mice. Two-way repeated measures ANOVA showed that THIP significantly reduced breathing rate, but that the effect depended on genotype (*P* = *0.037*, n = 6 per group). Post-hoc analyses identified a decrease in breathing rate of 19.9 ± 2.7% in the wild-type mice (*P* = *0.015*, Fig. 4c). In *Gabrd* ^−/−^ mice, however, THIP did not change breathing rate (*P* = *0.627*, Fig. 4c). Due to the low number of experiments (n = 6 per group), we performed additional Mann-Whitney Rank Sum non-parametric tests and showed that breathing rate was decreased by THIP in wild-type (*P* = *0.041*), but not in *Gabrd* ^−/−^ mice (*P* = *0.485*). The data also showed that there was no effect of genotype on the response of diaphragm amplitude to systemic injection of THIP (both *P* > *0.688*, two-way repeated measures ANOVAs, Fig. 4d). A Mann-Whitney test also showed no significant differences between the two groups (*P* = *0.394*). These results further support the idea that THIP reduces rhythmic breathing without affecting motor amplitudes by specifically activating δGABA_A_Rs.

### State-dependent regulation of respiratory network activity by δGABA_A_Rs

**Figure 5:**
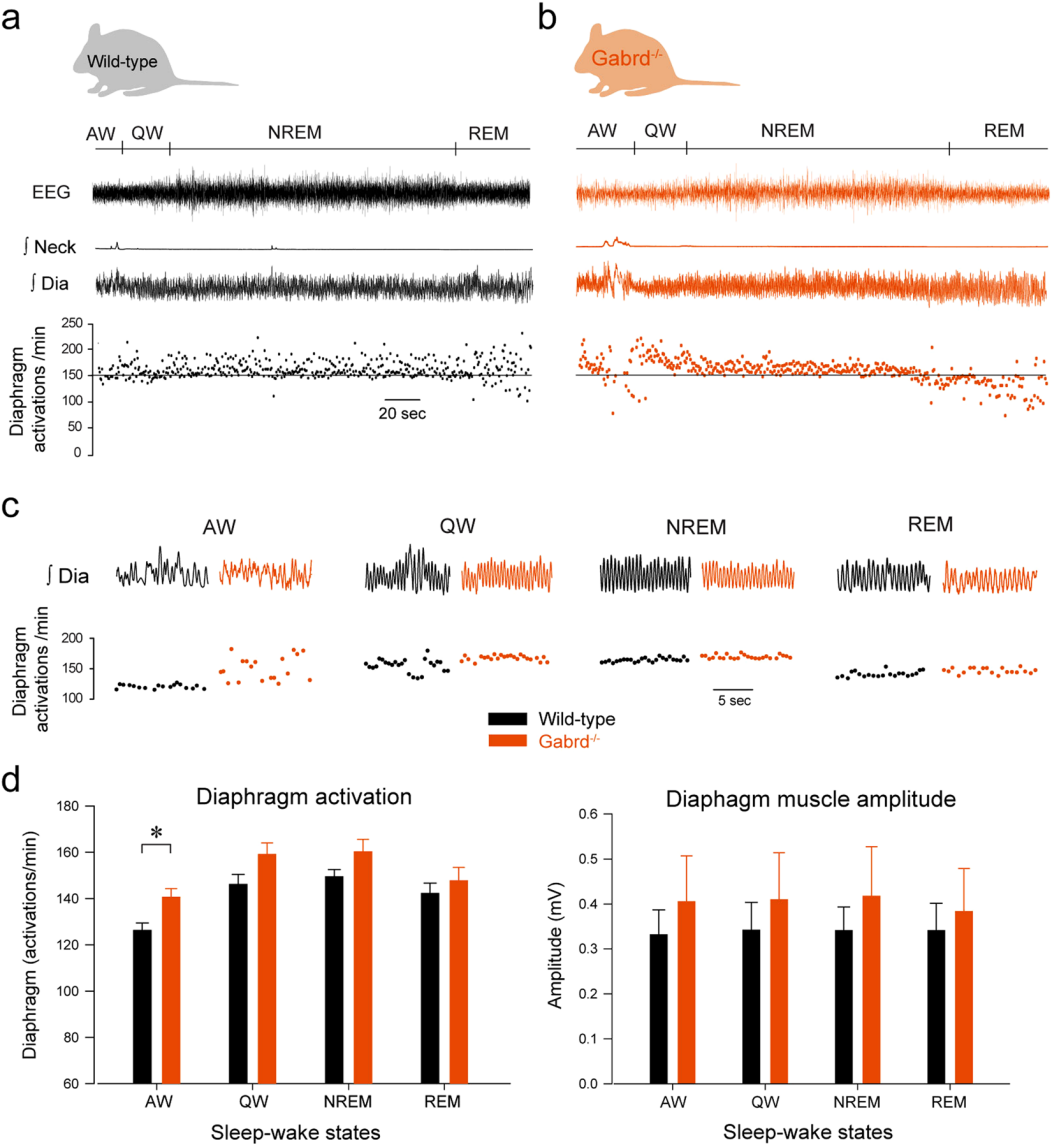
δGABA_A_R regulate diaphragm muscle activity during active wakefulness but not in quiet wakefulness and sleep. In freely behaving adult mice, diaphragm rhythmic activity (numbers of activations per minute) was increased in *Gabrd* ^−/−^ compared to wild-type mice (**a–c**) in active wakefulness (AW), but not in quiet wakefulness (QW), non-rapid eye movement (NREM) sleep or rapid eye movement (REM) sleep. There was no differences in diaphragm amplitude between wild-type and *Gabrd* ^−/−^ mice at each states (**d**). Data are indicated as mean ± SEM. *Indicates mean data significantly different with *P* < *0.05*. Dia, diaphragm muscle. Neck, neck muscle. EEG, electroencephalography.

Under anesthesia, the frequency of diaphragm muscle activations corresponds to the frequency of breathing, because rhythmic breathing is autonomously generated by the respiratory network and is not influenced by non-respiratory or behavioural activations^28,29^. To determine whether the frequency of diaphragm muscle activations is influenced by δGABA_A_Rs in freely-behaving animals, we measured respiratory muscle activities in non-anesthetized wild-type and *Gabrd* ^−/−^ mice (Fig. 5a,b). We first found that the effect of genotype on the frequency of diaphragm muscle activations depended upon the prevailing sleep-wake state (*P* = *0.039*). During active wakefulness, *Gabrd* ^−/−^ mice exhibited an increased frequency of diaphragm muscle activations compared to wild-type mice (*P* = *0.005*, Fig. 5c,d), but no differences were observed in quiet wakefulness, NREM and REM sleep (*P* > *0.235*, Fig. 5d). The averaged *amplitude* of diaphragm activity was not statistically different between the wild-type and *Gabrd* ^−/−^ mice (*P* = *0.587*) and there was no effect of genotype that depended upon sleep-wake state (*P* = *0.639*, Fig. 5d). These data suggest that a δGABA_A_R mechanism controls the rhythmic components of respiratory network activity rather than the magnitude of the oscillatory motor output.

**Figure 6:**
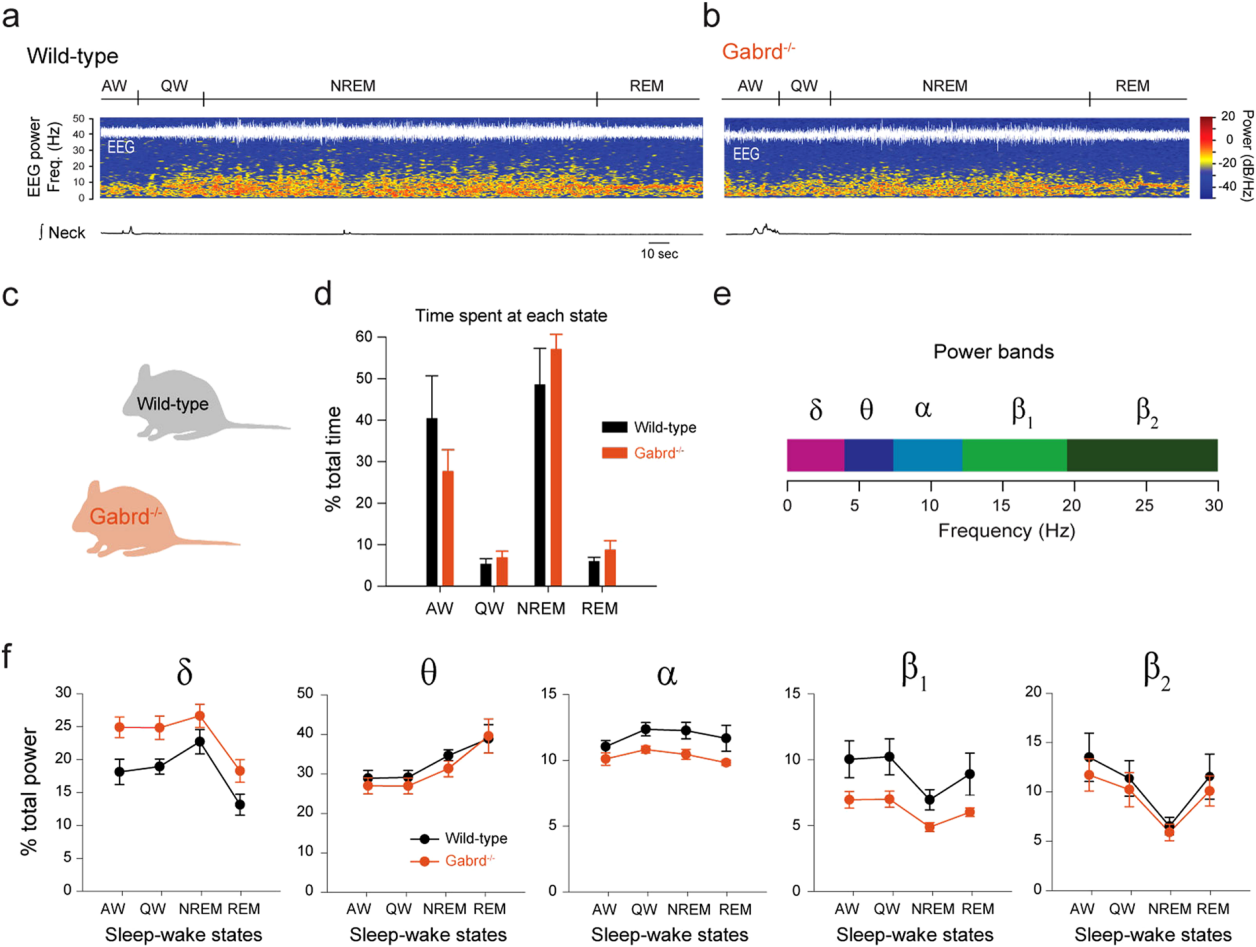
Sleep architecture and cortical changes in *Gabrd* ^−/−^ compared to wild-type mice. Spectrogram, electroencephalography activity in wild-type (**a**) and *Gabrd* ^−/−^ mice (**b**). *Gabrd* ^−/−^ mice (**c**) showed the same amount of time spent at each sleep-wake state than *Gabrd* ^−/−^ mice (**d**). Electrocortical band powers (**e**) were quantified for wild-type and *Gabrd* ^−/−^ mice for each sleep-wake state. *Gabrd* ^−/−^ mice showed similar power bands than wild-type mice (**f**). Data are presented as mean ± S.E.M. AW, active wakefulness, QW, quiet wakefulness, NREM, non-rapid-eye movement sleep. REM, rapid-eye movement sleep. EEG, electroencephalography.

We then determined whether the lack of δGABA_A_Rs in *Gabrd* ^−/−^ mice simply increases overall activity, i.e. the animal is more active. We found that *Gabrd* ^−/−^ mice exhibited similar amounts of time spent in each sleep-wake state compared to wild-type mice (*P* = *0.384*, n = 7 per group, Fig. 6a-d). *Gabrd* ^−/−^ mice also exhibited a similar overall EEG frequency spectrum (Fig. 6e) compared to wild-type mice. There was no main effect of genotype on EEG frequencies in the δ, θ, and β_2_ frequency bands (each *P* > *0.156*, n = 7 per group, Fig. 6f), whereas the effect in the β_1_ band was at the threshold for significance (*P* = *0.050*), and α activity was significantly lower in the *Gabrd* ^−/−^ mice (*P* = *0.038*). There was no effect of genotype on EEG activity in any frequency band that depended upon the prevailing sleep-wake state (all *P* > *0.440*). Overall, these data show that, in the *Gabrd* ^−/−^ mice, the increased frequency of diaphragm muscle activations in active wakefulness (Fig. 5c) was due to effects of altered δGABA_A_R-mediated inhibition during active behaviors.

### Behaviour-dependent modulation of respiratory network activity by δGABA_A_Rs

**Figure 7:**
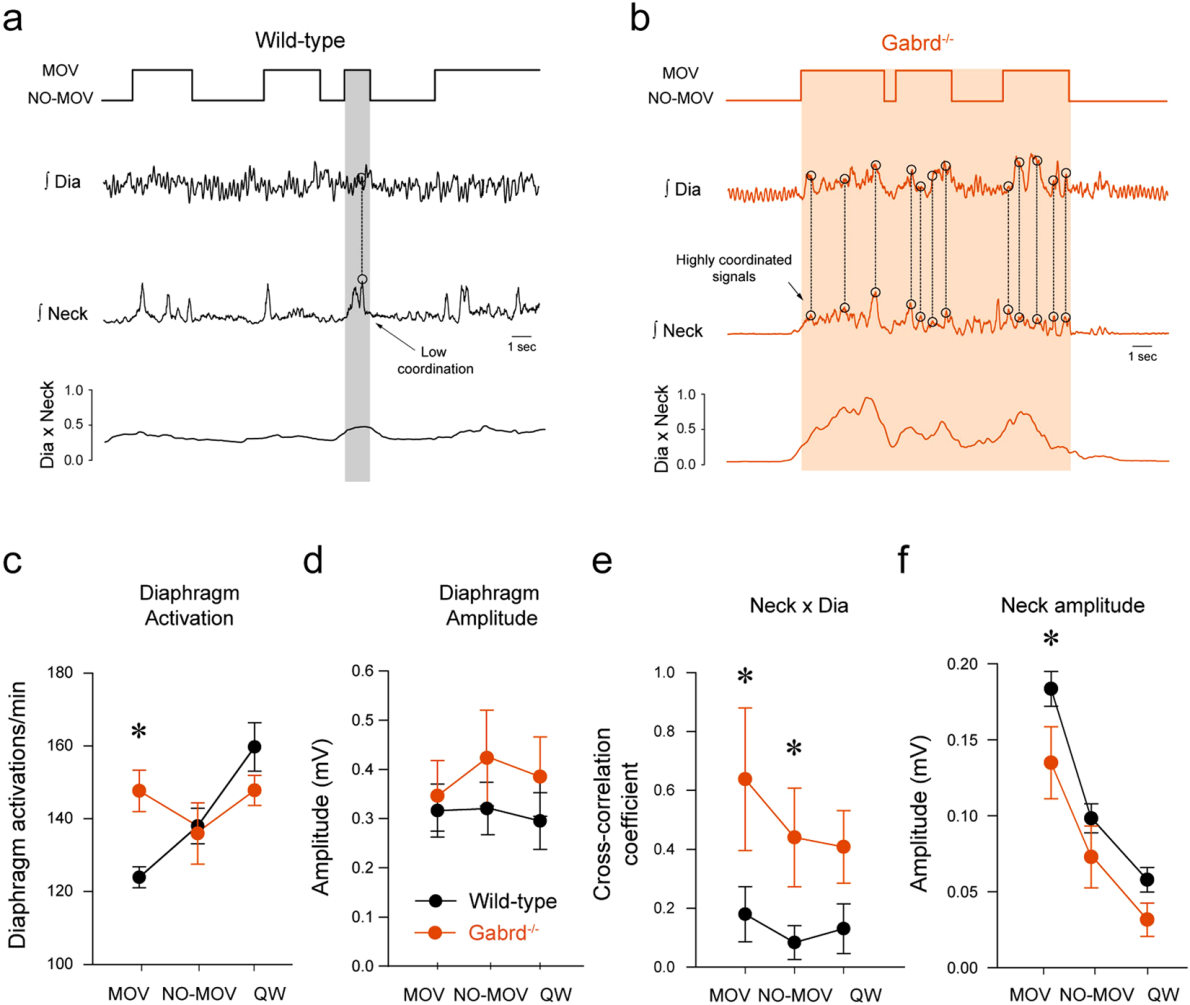
Synchronization of diaphragm and neck muscle activities during active behaviours in the absence of δGABA_A_R. In wild-type mice during movements and active wakefulness, diaphragm and neck muscle were not synchronized (**a**), whereas they were highly synchronized in *Gabrd* ^−/−^ mice (**b**). Diaphragm muscle activity was significantly higher in *Gabrd* ^−/−^ than in wild-type mice during movements (MOV) but not during active wakefulness without movements (NO-MOV) and quiet wakefulness (n = 7, **c**). Diaphragm amplitude did not differ between wild-type and *Gabrd* ^−/−^ mice (**d**). Cross-correlation indexes between diaphragm and neck muscle activities were significantly higher in *Gabrd* ^−/−^ during movements and active wakefulness without movements (**e**). Neck muscle amplitude was higher in wild-type than in *Gabrd* ^−/−^ mice during movements (**f**) *indicate values significantly different between wild-type and *Gabrd* ^−/−^ mice with *P* < *0.05*. MOV, active wakefulness with movements. NO-MOV, active wakefulness without movements. QW, quiet wakefulness. Dia, diaphragm muscle activity. Neck, neck muscle activity. Dia x Neck, cross-correlation between diaphragm and neck muscle activities. *Gabrd* ^−/−^, δ-subunit knockout mice.

The above data showed that reduced δGABA_A_R-mediated tonic inhibition led to increased diaphragmatic rhythmic activity (Fig. 5a). We then performed additional analyses of these signals in wakefulness to further stratify the effects by behaviors (Fig. 7a,b). The effect of genotype on the frequency of diaphragm muscle activations depended upon the type of behavior exhibited during wakefulness (*P* = *0.003*, n = 7 in each group, 2-way repeated measures ANOVA). Post-hoc analysis identified that during MOV, the frequency of diaphragm activations (i.e., the combination of behavioral and respiratory activations) was significantly higher in the *Gabrd* ^−/−^ mice compared to the wild-type mice (*P* = *0.006*, Fig. 7c). This effect was specific to MOV because this difference between the *Gabrd* ^−/−^ mice and the wild-types did not occur during NO-MOV (*P* = *0.801*) or quiet wakefulness (*P* = *0.147*). In contrast, there was no effect of genotype on diaphragm amplitude that depended upon the type of behavior exhibited during wakefulness (*P* = 106). We also observed that neck muscle activity was lower in the *Gabrd* ^−/−^ mice compared to wild-type mice. These additional analyses, however, identified that this genotype effect on neck muscle activity was confined to periods of MOV (*P* = *0.001*, Fig. 7e **)**, and not NO-MOV or quiet wakefulness (both *P* > *0.147*). These data further suggest that a δGABA_A_R-mediated mechanism modulates the rhythmic components of respiratory network activity rather than the magnitude of the oscillatory motor output.

### δGABA_A_Rs and respiratory activity during active wakefulness associated with movements

To identify whether the increased in diaphragm activation observed above is due to behavioural control of respiratory circuits, we tested the degree of coupling between diaphragm muscle and postural motor activities, two motor systems controlled by different neural circuits, across the behavioural states, and tested for an influence of δGABA_A_R-mediated modulation of respiratory network activity. Accordingly, using cross correlation, we quantified the degree to which the diaphragm and postural (neck) muscles are synchronized across MOV, NO-MOV and quiet wakefulness in the wild-type and *Gabrd* ^−/−^ mice. In the *Gabrd* ^−/−^ mice, the diaphragm and neck muscle activities showed distinct activations with multiple periods of coordination and synchronization during periods of MOV compared to the wild-type mice, (Fig. 7a,b). The group data confirmed that the effect of genotype on the synchronization of diaphragm and neck muscle activations depended upon the type of behavior exhibited during wakefulness (*P* = *0.006*, n = 7 in each group, 2-way repeated measures ANOVA). Post-hoc analyses showed that diaphragm and neck muscle activities were highly synchronized during MOV in the *Gabrd* ^−/−^ mice but were not in the wild-type mice (*P* = *0.006*, Fig. 7e). In NO-MOV, the diaphragm and neck muscle activations were also synchronized in *Gabrd* ^−/−^ but less so in wild-type mice (*P* = *0.029*, Fig. 7f). Together, these results indicate that the absence of δGABA_A_R-mediated tonic inhibition leads to more synchronization between diaphragm and postural (neck) motor activities during periods of active wakefulness associated with movements.

## Discussion

Post-synaptic inhibition, such as GABA_A_ receptor-mediated inhibition, is important to modulate rhythm in many neural circuits. Synaptic GABA_A_ receptors are implicated in respiratory control and are present throughout the respiratory network^30^, but the role of extra-synaptic GABA_A_ receptors is unknown. Here, we studied whether extrasynaptic δGABA_A_Rs provide a level of tonic inhibition in the respiratory network to modulate diaphragm rhythmic activity and to dampen rhythmicity during active motor behaviours. Using a combination of pharmacological and genetic approaches, we found that δGABA_A_Rs in the preBötC region function to decrease respiratory network activity. We then identified that the absence of δGABA_A_Rs in the *Gabrd* ^−/−^ mice increases respiratory network activity and motor activities during wakefulness when compared to wild-type mice. In *Gabrd* ^−/−^ mice, diaphragm activity is also synchronized to locomotor activity, an effect less pronounced in wild-type mice. The presence of an abnormal number of behavioral components in diaphragm muscle activity observed in *Gabrd* ^−/−^ mice suggests that δGABA_A_Rs may dampen the influence of non-respiratory behaviors on respiratory network activity to maintain breathing.

### GABA and the respiratory network

In the ventrolateral medulla of adult rats, glutamic acid decarboxylase immunoreactivity, a marker of GABAergic cells, has been identified in post-inspiratory and inspiratory neurons (Okazaki *et al*., 2001). In the preBötzinger Complex, GABA_A_ receptors are present in inspiratory neurons and non-respiratory neurons^7^, suggesting that GABA in the preBötC may modulate inspiratory activity but also non-respiratory activity. GABA_A_ receptor stimulation at the preBötC can depress breathing whereas antagonism has little or no effect^9,10^, suggestive of a GABA_A_ receptor-mediated inhibition that shapes respiratory motor outputs rather than generate respiratory rhythm *per se* ^11^. Although GABA_A_ receptors mainly exert their effects by inducing fast post-synaptic hyperpolarization, GABA also activates extra-synaptic GABA_A_ receptors located outside the synapse and generates tonic inhibition to reduce network excitability^13^. Our data indicate that the δGABA_A_R-preferring agonist THIP perfused in the ventrolateral medulla decreased breathing rate, at least in great part, by acting at the preBötC level. To confirm the site of action of THIP and to demonstrate that THIP modulates neurons in the vicinity of the preBötC, we showed that when THIP was perfused close to the preBötC, it induced a faster and more pronounced decrease in breathing rate than when it is perfused away from it. These relationships were supported by correlations between the latencies or magnitudes of THIP effect and distances from perfusion site to the preBötC as previously described and validated^19,20,24^. Although THIP is a δGABA_A_R-preferring agonist^27^, we further verified that THIP selectively targets δGABA_A_Rs by using *Gabrd* ^−/−^ mice that lack these receptors. The absence of response to THIP in *Gabrd* ^−/−^ mice further identified that selective activation of δGABA_A_Rs underlie inhibition of respiratory network activity by THIP. The combination of these two approaches support the idea that δGABA_A_Rs in the region of the preBötC have the capacity to modulate respiratory network activity.

### Respiratory network excitability

The respiratory network, and at its core the preBötC, form a dynamic network endogenously generating rhythmic motor activity. To spontaneously generate respiratory motor rhythm, the network needs a sufficient level of excitability^31^. *In vitro*, when the respiratory network is isolated and network excitability is reduced, potassium concentration is typically raised to increase excitability and so generate rhythmic activity. In addition, respiratory network excitability is modulated by G-protein-coupled receptors such as µ-opioid receptors, where their activation at the preBötC inhibits breathing rate^19^, an effect not observed in mice lacking functional G-protein-inwardly rectifying potassium (GIRK) channels^22^. Inhibition through GIRK channels likely involves a network level modulation^32^. Such large scale effect of neurotransmitters is known as volume transmission and δGABA_A_Rs reduce network excitability through this mechanism^33^. Volume transmission, as opposed to wiring transmission, modulate network excitability via neurotransmitters diffusing into the extracellular space and then by activating extra-synaptic receptors^14^. Although this type of neurotransmission is slower than wiring transmission, it is sufficiently fast to modulate the respiratory network and ongoing motor systems^27^. Volume transmission and GABAergic inhibition through δGABA_A_Rs may therefore be a potential mechanism modulating network excitability. Here, we identify, for the first time, the functional role of δGABA_A_Rs, which are uniquely expressed in the extrasynaptic space, in the preBötC region and the capacity of these receptors to modulate respiratory network activity during active behavior.

### Respiratory network behaviour and extra-synaptic GABA_A_ receptors

In states of reduced arousal, i.e. anesthesia, when behaviour is suppressed and the respiratory network is autonomous, activation of δGABA_A_Rs at the preBötC significantly reduced respiratory network activity. Although these data demonstrate that δGABA_A_Rs in the preBötC region have the capacity to modulate respiratory network activity, the role of δGABA_A_Rs in response to endogenous GABA release cannot be assessed due to the lack of a selective δGABA_A_R antagonist. To identify the endogenous role of δGABA_A_Rs in modulating respiratory network activity, we compared diaphragm muscle activity (a surrogate of respiratory network activity) between wild-type and *Gabrd* ^−/−^ mice at various states of arousal and during behaviours. Although δGABA_A_Rs did not play a significant role in modulating respiratory network activity during sleep and quiet wakefulness, rhythmic activity of the diaphragm muscle was higher in *Gabrd* ^−/−^ than in wild-type mice in active wakefulness, especially during movements. *Gabrd* ^−/−^ mice also showed synchronized diaphragm and neck muscle activities during movements, pointing out that, without δGABA_A_Rs, non-respiratory behaviours greatly influenced respiratory network activity. Although the mechanisms underlying the role of δGABA_A_Rs in inhibiting respiratory network activity only during movements have not been yet identified, our data suggest that δGABA_A_Rs play an endogenous role in the modulation of respiratory network activity, especially during active non-respiratory behaviors.

### Regulation of network excitability by tonic inhibition

Extra-synaptic δGABA_A_Rs play a significant role in modulating respiratory activity during conscious behaviors *in vivo* when the levels of excitation independent of breathing *per se* is high^28,29^. Excitatory inputs from descending motor pathways may be inhibited by δGABA_A_Rs to maintain the essential autonomic function of breathing while non-respiratory behaviours are performed. The notion of tonic inhibition regulating network excitability has been demonstrated in thalamo-cortical networks^26^, in the hippocampus^34^, and in the cerebellum^35^. Our findings identify that δGABA_A_Rs modulate rhythmic breathing, and dampen behavioral influences on rhythmic motor activity during active behaviors *in vivo*. These results identify a previously unrecognized role of δGABA_A_Rs in modulating the endogenously active respiratory network *in vivo*, the persistence and stability of which is critical to homeostasis. Identification of the role of extrasynaptic GABA_A_ receptors provides a better understanding of how breathing is affected by changes in extrasynaptic GABA_A_ receptor expression and function under pathological conditions such inflammation^36^ and exposure to anesthetics and neurosteroids^13,18^.
